# The effect of vitamin D treatment on quality of life in patients with fibromyalgia

**DOI:** 10.1007/s11845-023-03521-4

**Published:** 2023-09-14

**Authors:** Sedef Ersoy, Fatma Nur Kesiktas, Busra Sirin, Derya Bugdayci, Nurdan Paker

**Affiliations:** Istanbul Fizik Tedavi Rehabilitasyon Egitim ve Arastirma Hastanesi, Istanbul, Turkey

**Keywords:** Fibromyalgia, Fibrositis, Muscular, Rheumatism, Vitamin D

## Abstract

**Background:**

Fibromyalgia is a syndrome characterized by chronic widespread pain accompanied by fatigue, disrupted sleep quality, cognitive impairments, subjective soft tissue swelling, and somatic symptoms. There are conflicting results in the literature regarding the prevalence of vitamin D deficiency in fibromyalgia patients and the reduction of symptoms after supplementation.

**Aims:**

Our study aims to evaluate the effectiveness and reliability of vitamin D supplementation in patients diagnosed with fibromyalgia.

**Methods:**

In our cross-sectional clinical study, 180 female patients aged 18 to 65 diagnosed with fibromyalgia according to the 2010 American College of Rheumatology Diagnostic Criteria were included. Oral vitamin D3 replacement of 50,000 IU was administered for 12 weeks. Patients' Fibromyalgia Impact Questionnaire (FIQ)and Visual Analogue Scale (VAS) scores were evaluated before and after the study.

**Results:**

Significant differences were observed in the FIQ scores of the 180 fibromyalgia patients before and after vitamin D supplementation (p < 0.05). There was also a significant improvement in VAS scores (p < 0.01). A negative correlation between vitamin D and VAS as well as FIQ scores was found in the study.

**Conclusion:**

We determined that vitamin D deficiency is significantly more prevalent in patients diagnosed with fibromyalgia. Vitamin D supplementation was observed to have a positive effect on quality of life and reduction of pain.

## Introductıon

Fibromyalgia is a syndrome characterized by chronic widespread pain accompanied by fatigue, decreased sleep quality, cognitive impairments, subjective soft tissue swelling, and somatic symptoms [1]. In addition to the pain felt in the muscles and skeletal system for at least 3 months, the presence of tender points on examination is important for diagnosis. The prevalence of the disease increases between the ages of 40 and 60, with the female gender comprising 85–90% of patients [2]. Although its etiology is not fully understood, genetic, neurohormonal, and psychosocial factors are thought to play a role in its development [3]. Physical and emotional stress, as well as cold weather, can provoke symptoms [4]. The treatment of fibromyalgia involves a multidisciplinary approach, including lifestyle modifications, medical treatment, nutritional support, and cognitive-behavioral therapies [5]. Serotonin-norepinephrine reuptake inhibitors (SNRIs) and antiepileptic drugs are commonly preferred medical agents for pharmacological treatment due to their side effect profile [6]. Non-steroidal anti-inflammatory drugs and opioid analgesics are also used for symptom control [7].

Vitamin D is a steroid hormone that regulates calcium and phosphate levels in the blood [8]. Its deficiency negatively affects calcium and phosphorus metabolism, osteoblastic cell activity, and bone mineral density [9]. Vitamin D deficiency has been associated with various pathologies such as chronic pain, delayed growth and development in children, malignancy, and autoimmune diseases [10]. It leads to symptoms like widespread chronic pain and fatigue, often coinciding with fibromyalgia syndrome (FMS). FMS, which is among the central sensitization syndromes, exerts its effects through inflammatory pathways and neurotransmitters, similar to the mechanism involved in vitamin D deficiency. It is known that vitamin D deficiency initiates inflammatory processes that lead to the development of chronic pain and alterations in sensitivity [11]. Furthermore, restricted physical activity levels and insufficient sunlight exposure due to depressive symptoms or severe pain levels in FMS patients are one of the reasons explaining the connection with vitamin D deficiency. In this context, vitamin D deficiency gains importance in differential diagnosis and treatment processes [12]. Moreover, it has been demonstrated that vitamin D deficiency in patients diagnosed with fibromyalgia is associated with impaired quality of life and increased perceived pain levels [13]. This suggests that evaluating vitamin D levels in FMS patients and replacing them when necessary could contribute significantly to symptom control and quality of life in the treatment process. Therefore, our study aims to determine the prevalence of vitamin D deficiency in FMS patients and investigate the effects of vitamin D replacement on chronic pain levels and quality of life in patients with insufficient vitamin D levels.

## Method

Our study was designed as a prospective cross-sectional clinical research. Research ethics committee approval (protocol no: 2023–287) was obtained from the Clinical Research Ethics Committee of Bakırköy Dr. Sadi Konuk Training and Research Hospital. A total of 180 female patients aged 18–70 with a diagnosis of fibromyalgia according to the American College of Rheumatology 2010 Diagnostic Criteria were included in the study. The exclusion criteria for our study were the presence of neurological, rheumatological, endocrine diseases, hepatic or chronic renal disease, cardiovascular diseases, malabsorption, history of malignancy, active infection, psychiatric illness, pregnancy and lactation period, and cognitive impairment.

After providing information about the research and obtaining informed consent from the patients, their demographic information, existing comorbidities, and medications used were questioned and recorded. During routine assessment, patients' 25-OH Vitamin D levels were examined, and based on their vitamin D levels, they were categorized as severe deficiency if below 10 ng/mL, vitamin D deficiency between 10–20 ng/mL, vitamin D insufficiency between 21–29 ng/mL, sufficient level (preferred range 40–60 ng/mL) if above 30 ng/mL, and vitamin D intoxication if above 150 ng/mL.

Blood samples were collected using gel separator biochemistry tubes from Becton Dickinson, and after clotting, serum was obtained by centrifugation at 4000 g for 10 min.

Patients with levels below 29 ng/mL were given 50,000 IU oral Vitamin D3 replacement weekly for 12 weeks. Before and after replacement, the Fibromyalgia Impact Questionnaire (FIQ), a Turkish version with proven validity and reliability, was applied to assess the impact on quality of life. FIQ consists of 10 questions that inquire about the level of impact on daily activities, fatigue, pain level, morning discomfort, and mood over the past week. The maximum score is 80, and the minimum score is 0 [14]. Pain assessment was done using the Visual Analog Scale (VAS). The visual analog scale (VAS) is a pain rating scale. Scores are based on self-reported measures of symptoms that are recorded with a single handwritten mark placed at one point along the length of a 10-cm line that represents a continuum between the two ends of the scale “no pain” on the left end (0 cm) of the scale and the “worst pain” on the right end of the scale (10 cm) [15].

## Statistical analysıs

Statistical analysis of the data was performed using SPSS (Statistical Package for the Social Sciences, Chicago, IL, USA) version 20.0. The normal distribution of the data was tested using the Shapiro–Wilk test. Descriptive statistics for quantitative data were presented as means, medians (minimum–maximum), and means ± standard deviation (SD). Qualitative data were expressed as numbers (n) and percentages (%). Pearson correlation was used for correlation analysis. The Student t-test was used for group comparisons. A significance level of p < 0.05 was considered statistically significant.

## Results

A total of 180 female patients aged 18–70 with a diagnosis of fibromyalgia were included in our study. The mean age of the patients was 42.77, and the mean 25-OH Vitamin D level was 20.32 (Table 1). When the age distribution was examined, 36% of the patients were in the 30–49 age range, and 22% were in the 50–59 age range, which is parallel to the age range where fibromyalgia syndrome is frequently observed (Table 2).

**Table 1:** Demographic Data

	n	Minimum	Maximum	Mean	Standard deviation
Age	180	**18**	**70**	**42,77**	**10,92**
25 0H D Vit	180	**3,0**	**65,71**	**20,32**	**14,61**
Disease duration	180	**4**	**32**	**11,75**	**11,92**

Minimum, maximum, standard deviation, and mean values are provided in the table

**Table 2:** Distribution of Patient Ages

			%	Cumulative Percent
Age	≤ 30	29	16,1	16,1
	31–39	32	17,8	33,9
	40–49	65	36,1	70,0
	50–59	40	22,2	92,2
	≥ 60	14	7,8	100,0
	Total	180	100,0	

When the patients included in our study were grouped according to their 25-OH Vitamin levels, it is observed that there were 57 patients with levels below 10 ng/mL, 61 patients in the range of 10–20 ng/dL, 22 patients in the range of 21–29 ng/dL, and 40 patients with levels above 30 ng/dL. Deficiency or severe deficiency of vitamin D levels was present in 65.6% of the patients, and 12.2% had vitamin D insufficiency. Consequently, it can be concluded that 77.8% of our patients were in need of vitamin D replacement (Table 3).

**Table 3:** 25-OH Vitamin D Levels

		N	%	Cumulative Percent
Vit D	** < 10 ng/mL**	57	31,7	31,7
	**10- 20 ng/mL**	61	33,9	65,6
	**21—29 ng/mL**	22	12,2	77,8
	** > 30 ng/mL**	40	22,2	100,0
		180	100,0	

When vitamin D levels were grouped according to age ranges, the rate of vitamin D deficiency and severe vitamin D deficiency in the patient group below 40 years of age was significantly higher compared to the patient group aged 40 and above (p < 0.0001*) (Table 4).

**Table 4:** Vitamin D Levels According to Age Groups

**Age**	**Vit D**	**f**	**%**	**Total %**	
** ≤ 30**	** < 10 ng/mL**	**14**	**48,3**	**48,3**	p < 0.0001*
	**10 - 20 ng/mL**	**12**	**41,4**	**89,7**	
	**21 - 29 ng/mL**	**2**	**6,9**	**96,6**	
	**30 ng/mL**	**1**	**3,4**	**100,0**	
	**Total**	**29**	**100,0**		
**31–39**	** < 10 ng/mL**	**13**	**40,6**	**40,6**	
	**10 - 20 ng/mL**	**12**	**37,5**	**78,1**	
	**21 - 29 ng/mL**	**2**	**6,3**	**84,4**	
	**30 ng/mL**	**5**	**15,6**	**100,0**	
	**Total**	**32**	**100,0**		
**40–49**	** < 10 ng/mL**	**16**	**24,6**	**24,6**	
	**10 - 20 ng/mL**	**22**	**33,8**	**58,5**	
	**21 - 29 ng/mL**	**11**	**16,9**	**75,4**	
	**30 ng/mL**	**16**	**24,6**	**100,0**	
	**Total**	**65**	**100,0**		
**50–59**	** < 10 ng/mL**	**11**	**27,5**	**27,5**	
	**10 - 20 ng/mL**	**10**	**25,0**	**52,5**	
	**21 - 29 ng/mL**	**5**	**12,5**	**65,0**	
	**30 ng/mL**	**14**	**35,0**	**100,0**	
	**Total**	**40**	**100,0**		
** ≥ 60**	** < 10 ng/mL**	**3**	**21,4**	**21,4**	
	**10- 20 ng/mL**	**5**	**35,7**	**57,1**	
	**21 - 29 ng/mL**	**2**	**14,3**	**71,4**	
	**30 ng/mL**	**4**	**28,6**	**100,0**	
	**Total**	**14**	**100,0**		

Percentages (%), frequencies (f), and the correlation coefficient (r = 0.284, p < 0.0001*) are presented. The correlation is statistically significant

Before treatment, the patients' pain levels were evaluated using the Visual Analog Scale (VAS). The mean initial VAS value was 7.69, which decreased to 5.14 after replacement. The mean FIQ score was 67.46 at the beginning and decreased to 53.65 after treatment (Table 5).

**Table 5:** Initial and Post-Treatment VAS and FIQ Scores

	Initial scores mean ± SD	After 12 weeks mean ± SD	
VAS	7.69 ± 1.23	5.14 ± 1.21	P < 0.01*
FIQ	67.46 ± 9.97	53.65 ± 8.95	P < 0.05*

*FIQ* Fibromyalgia Impact Questionnaire, *VAS* visual analog scale

## Dıscussıon

Fibromyalgia is one of the commonly observed chronic pain syndromes in society, significantly impacting the quality of life of patients. The prevalence of Vitamin D deficiency in FM patients, its relationship with symptom severity, and its effect on daily life have been frequently investigated in the literature, yielding conflicting results [10]. Vitamin D is considered essential for the human body, and its deficiency has been associated with chronic widespread pain syndromes, cardiovascular pathologies, autoimmune diseases, and certain types of cancer [16]. The primary aim of this study is to determine the frequency of vitamin D deficiency in the fibromyalgia patient group and to assess the level of reduction in pain severity and improvement in quality of life after replacement therapy.

In the study by Aktunç et al., vitamin D levels were evaluated in patients diagnosed with FMS and a healthy group. While the deficiency and insufficiency rate of vitamin D in the patient group was 80%, this rate was 85% in the healthy group, and it was not found to be statistically significant [10]. Similarly, in the study conducted by Doğru et al., fibromyalgia and healthy groups were examined, and vitamin D deficiency and insufficiency were detected in 60% of the patients and 50.7% of the control group [17]. In our study, we found that 65.6% of FMS patients had deficiency or severe deficiency in vitamin D levels, and 12.2% had vitamin D insufficiency. 77.8% of the patients required vitamin D replacement.

Yalçın et al. evaluated 80 female patients diagnosed with fibromyalgia and found that 57.5% of them had vitamin D levels lower than 20 ng/mL [18]. Other studies have also found lower vitamin D levels in the fibromyalgia group compared to the healthy group [19–21]. Souza et al. found that in 122 out of 593 fibromyalgia patients (20.6%), the vitamin D level was below 25 ng/dL [22]. Several studies in the literature confirm the relationship between chronic widespread musculoskeletal pain and 25 OH D vitamin deficiency [23–25]. Olama et al. examined 50 patients diagnosed with FMS and 50 individuals in a control group, and they found significantly lower vitamin D levels in the FMS group [26].

In the literature, there are studies that have found a negative correlation between vitamin D levels and pain scores, trigger point counts, and FIQ scores [16, 27]. In the study by Doğru et al., there was no correlation between vitamin D levels and VAS and FIQ scores, but significant changes were observed in VAS and FIQ scores before and after vitamin D replacement therapy [17]. In the study by Yalçın et al., the group with vitamin D levels below 20 ng/mL had significantly higher FIQ and VAS scores [18]. Souza et al. also found significantly higher FIQ scores in the group with vitamin D deficiency [22]. Consistent with the literature, our study also revealed a significant decrease in VAS and FIQ scores after 12 weeks of 50,000 IU vitamin D replacement therapy. In contrast, Plata et al. stated that a 12-week vitamin D replacement did not significantly alter VAS and FIQ scores [28]. Similarly, Warner et al. reported in their study that vitamin D replacement did not lead to significant changes in pain scores [29]. In the study by Wepner et al., they included 30 female patients diagnosed with FMS and found significant improvement in pain scores after vitamin D replacement, but no significant change in FIQ scores [30]. Warner et al. used ergocalciferol (vitamin D2) for replacement, while Wepner et al. used cholecalciferol (vitamin D3) replacement. Pre- and post-replacement measurements showed that cholecalciferol was more effective in increasing vitamin D levels. Therefore, the choice of vitamin D type might contribute to the differences in results [15]. Similarly, in our study, cholecalciferol replacement was administered, following the approach of the study by Wepner et al.

In the study by Mirzaei et al., similar to our research, the addition of vitamin D to medical treatment in FMS patients was found to have a positive effect on improving quality of life and reducing pain severity [31]. In the study by Armstrong et al., they observed significantly higher levels of anxiety and depression in FMS patients. They emphasized that anxiety and depression might result from immobilization, difficulty in maintaining regular aerobic exercise, and indirectly, inadequate exposure to sunlight [32]. Similarly, in the study by Al Allaf et al., they found lower vitamin D levels in FMS patients compared to the control group and suggested that this might be related to physical inactivity and insufficient sunlight exposure [33]. In our study, we also consider that the measurement of Vitamin D levels in winter months could contribute to the lower vitamin D levels. However, the absence of a control group and the inability to make direct comparisons are limitations of our study.

Huisman et al.'s study examined 25 systemic lupus erythematosus (SLE) patients and 25 FMS patients. They found that the vitamin D levels in SLE patients were expected to be lower due to sun avoidance but were statistically similar between the two groups. This study suggests a predisposition to vitamin D deficiency in FMS patients as well and supports other studies in the literature [34].

Armstrong et al. did not find a significant relationship between FIQ scores and vitamin D levels in their study [32]. In the study by Al-Allaf et al., while FIQ scores were found to be lower in the control group compared to the FMS group, there was no significant difference in FIQ scores between FMS patients with vitamin D deficiency and those without deficiency [33]. Similarly, there are other studies in the literature that couldn't find a relationship between vitamin D deficiency and FIQ scores [19, 35]. In contrast, in our study, significant changes were observed in VAS and FIQ scores when comparing the same group of patients with low and high vitamin D levels.

In our study, the prevalence of vitamin D deficiency was found to be significantly higher in patients under 40 years of age. Zuberi et al.'s study also found a lower average age in the group with vitamin D deficiency [36]. Plotnikoff et al. also reported in their research that 55% of patients with severe vitamin D deficiency were under the age of 30 [37]. Our study is in line with the literature in this regard.

In a study that examined chronic widespread musculoskeletal pain and FMS in relation to vitamin D deficiency in males, a significant association was found between pain and vitamin D levels. Moreover, this relationship persisted even when factors influencing pain such as physical activity level, BMI, smoking, and alcohol use were eliminated. This confirms that vitamin D deficiency has an independent relationship with chronic pain. Additionally, it indicates that this effect is not limited to the female gender [38]. One of the limitations of our study is the exclusion of male FMS patients. The absence of a placebo group in our study makes it difficult to objectively evaluate the positive results obtained. Therefore, comprehensive studies are needed that include placebo-controlled comparisons within the FMS patient group and with healthy volunteers, in order to better understand these findings.

## Conclusıons

Vitamin D deficiency is a common issue in society that significantly affects the lives and functional status of patients. The purpose of this study was to evaluate the impact of vitamin D levels on FMS patients. In our study, we observed a significant reduction in pain and FIQ scores after replacement therapy in FMS patients with accompanying vitamin D deficiency. This research highlights the importance of regularly measuring 25(OH)D levels and considering replacement therapy as a valuable adjunct treatment option in the management of FMS patients. All data utilised in this article are available to access.                                                                                                                          
